# Effect of Contact Pressure on Strain Distribution during Compression-Type Bulk Forming Processes

**DOI:** 10.3390/ma16145041

**Published:** 2023-07-17

**Authors:** Joong-Ki Hwang

**Affiliations:** School of Mechatronics Engineering, Korea University of Technology and Education, Cheonan 31253, Republic of Korea; jkhwang@koreatech.ac.kr; Tel.: +82-041-560-1642

**Keywords:** rolling, contact pressure, compression-type bulk forming, strain distribution

## Abstract

Inhomogeneity of the material properties of workpieces developed during compression-type bulk forming processes (CBFPs) is an important issue. The effect of contact pressure on the workpiece surface on the strain inhomogeneity in the workpiece was investigated to understand and reduce the formation of strain inhomogeneity during CBFPs. Workpieces fabricated via rod caliber rolling, rod flat rolling, plate flat rolling, and rod compression were analyzed and compared. The extent of strain inhomogeneity in a workpiece differs with the forming process, because the occurrence of macroscopic shear bands (MSBs) is dependent on the workpiece shape and tool design. A flat-rolled rod exhibits the maximum strain inhomogeneity, whereas a flat-rolled plate shows the minimum strain inhomogeneity. The occurrence of MSBs was influenced by the distribution of the normal contact pressure or compression stress. The MSBs were stronger when the contact pressure was higher in the edge region of the surface. For example, the flat-rolled plate exhibited weak MSBs due to the relatively uniform or higher contact pressure on the central region. In contrast, strong MSBs appeared in the flat-rolled rod and compressed rod, because the contact pressure in the edge region of these two processes was high. Thus, the strain inhomogeneity in a workpiece fabricated via CBFPs can be reduced by controlling the contact pressure distribution on the workpiece surface.

## 1. Introduction

**Figure 1:**
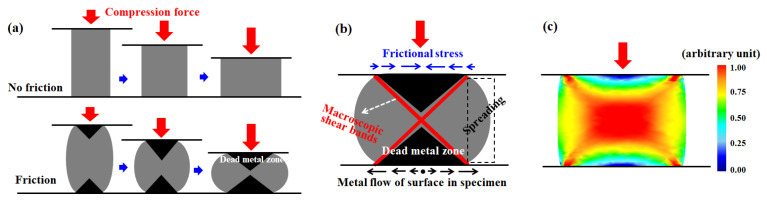
(**a**,**b**) Schematics of the effect of friction at the workpiece–tool interface and (**c**) distribution of effective strain during uniaxial compression.

In the metal-forming industry, compression-type bulk forming processes (CBFPs) are used extensively owing to their high formability and productivity. However, the inhomogeneity of the material properties of the product, that is, macroscopic shear bands (MSBs), typically develops in the process [1]. In this study, the MSBs are associated with the regions of the specimen with higher effective strain compared with other regions of the specimen. The MSBs and/or strain inhomogeneity during the CBFPs is primarily caused by the restricted material flow in the workpiece–tool interface because of the frictional stress, as schematically shown in Figure 1, and it depends on both the process conditions and the material properties [2,3,4,5,6,7,8]. 

Therefore, the strain inhomogeneity in a workpiece has been investigated during CBFPs, such as compression [2,9,10], side pressing [1,3], and rod flat rolling [11,12]. Eom et al. [9] reported that the strain distribution of a specimen was more homogeneous when increasing the strain hardening rate of material during compression based on the comparison of steels with high and low strain hardening rates. They explained this phenomenon in terms of the capability of the strain distribution in metals: a metal with high strain hardening rate can transfer the plastic region far away from the initial deformation regions, i.e., workpiece and tool interface. Kazeminezhad et al. [11] revealed the formation of MSBs during rod flat rolling using the finite and slab element method and validated the results based on the Vickers hardness test and metallographic examination of the cross-section of the flat-rolled copper rod. They showed that the strain inhomogeneity in the flat-rolled rod increased with an increasing friction factor and decreasing reduction ratio. In addition, Hwang [12] reported a very different behavior of hardness, strain, and twinning of flat-rolled rod with a region based on experimental testing and numerical simulation using twinning-induced plasticity (TWIP) steel. He revealed that the center region tended to have the maximum twin density, effective strain, and hardness, whereas the free surface region had the minimum values. Paul et al. [2] reported that the mechanism of MSB formation depended on the crystallographic orientation of material based on the plane strain compressed test using fine-grained aluminum. They showed that the strain-induced rotation of the crystal lattice led to the formation of specific MSBs and they facilitated slip propagation across the grain boundaries. Semiatin et al. [3] revealed that the tendency to form shear bands depends on test temperature and deformation based on an isothermal side pressing test using Ti-6A1-2Sn-4Zr-2Mo0.1Si alloy with a variety of working temperatures and deformation rates. 

The issue of strain inhomogeneity of a product becomes significant during the plastic forming process, as the demand for non-heat-treated products is increasing in metal-forming and related industries [13,14]. By eliminating heat treatments of products during the manufacturing process, several advantages can be obtained industrially as follows: production costs can be reduced, distortion of materials occurring during heat treatment can be prevented, production time can be reduced, and environmental pollution can be prevented. The strain inhomogeneity of a workpiece is significant in TWIP steels because they are used as the final products with non-heat treatments after the metal-forming process [15]. Therefore, decreasing the stain inhomogeneity in a workpiece during CBFP is essential to improve the quality and reduce the cost of the final product.

Recently, Hwang [16] reported that the homogeneity of the strain distribution in a workpiece tends to improve with increasing elongation and decreasing pure spreading during CBFP. Based on this, they suggested that low pure spreading and high elongation of a workpiece guarantee a more homogeneous and high-quality product during CBFP. Furthermore, the distribution of the contact pressure (*P*_c_) on the workpiece surface should affect the formation of MSBs as well as the roll force and tool wear [17,18,19,20]. However, to the best of the author’s knowledge, studies regarding the effect of the *P*_c_ on the MSBs of a material during CBFP are few. In particular, studies regarding the effect of the *P*_c_ on the MSBs of a material during the rolling process are rarely performed. Li et al. [19] reported that the distribution of effective strain in flat-rolled plate was more homogeneous when friction hill type *P*_c_ was formed during plate flat rolling compared with the formation of two-peak type *P*_c_. However, they did not reveal the influence of the *P*_c_ on strain inhomogeneity or MSB formation during the rolling process. Thus, it can be concluded that there is still a lack of understanding of the behavior of MSBs with the distribution of *P*_c_ during CBFP.

Therefore, this study evaluates the effect of the *P*_c_ on the strain inhomogeneity in a workpiece to reveal the mechanism of MSB formation and alleviate the strain inhomogeneity in the workpiece during CBFP. For a comprehensive study, four different CBFPs, i.e., rod caliber rolling, rod flat rolling, plate flat rolling, and rod compression, were analyzed and compared via finite element analysis (FEA) and experiments using a TWIP steel. FEA was used to evaluate the complicated distributions of strain, stress, and *P*_c_ in the workpiece during the process, and hardness tests were carried out to analyze the strain distribution throughout the surface region of the workpieces.

## 2. Experiment and Deformation Analysis

### 2.1. Experiments

A TWIP steel ingot weighing 50 kg was cast using vacuum induction. The chemical composition, as determined via spark optical emission spectrometer, was Fe-19.94Mn-0.6C-1.03Al (wt. %). The structure of the cast-ingot with a thickness of 125 mm was homogenized in a box furnace for 12 h at a furnace temperature of 1200 °C to reduce the segregation of chemical composition. The cast-ingot was then rolled to a plate with a thickness of 20 mm using several rolling machines at the final temperature of 950 °C, followed by air cooling at an ambient temperature of 20 °C.

**Figure 2:**
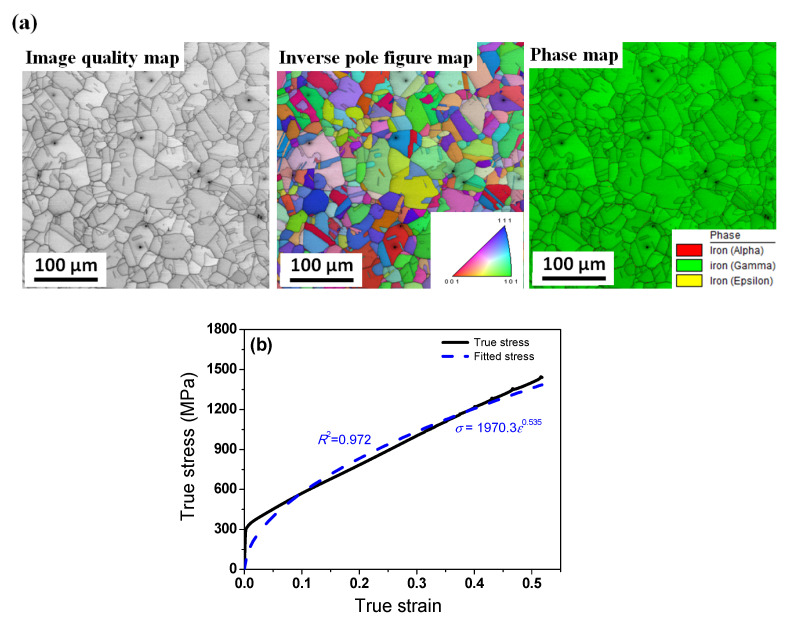
(**a**) Microstructure and (**b**) true tensile curve of hot-rolled TWIP steel.

Figure 2a represents the microstructure of the hot-rolled specimen using electron backscatter diffraction (EBSD). The specimen for EBSD observation was ground by mechanical polishing using papers with silicon carbide abrasive and diamond pastes from 6 to 1 μm. Colloidal silica suspensions were then applied to remove the residual stress around the surface of the specimen. EBSD maps of 400 μm × 400 μm were measured using a step size of 0.3 μm with a field emission scanning electron microscope, which is equipped with a TSL data acquisition system. The specimen’s tilting angle was approximately 70° and the acceleration voltage was 20 kV. The acquired EBSD data were analyzed using the commercial orientation imaging microscopy software. Based on the inverse pole figure map obtained via EBSD, recrystallized grains with annealing twins were seen, but deformation twins were not detected, as shown in Figure 2a. The calculated grain size was approximately 28 μm on average. Tensile test specimens (gauge diameter of 5 mm and length of 25 mm) were extracted along the rolling direction from the hot-rolled plate. The specimens were elongated at a slow strain rate of 10^−3^ s^−1^ at room temperature (RT, 26 °C). Figure 2b shows the true stress–strain curve of the specimen. 

**Figure 3:**
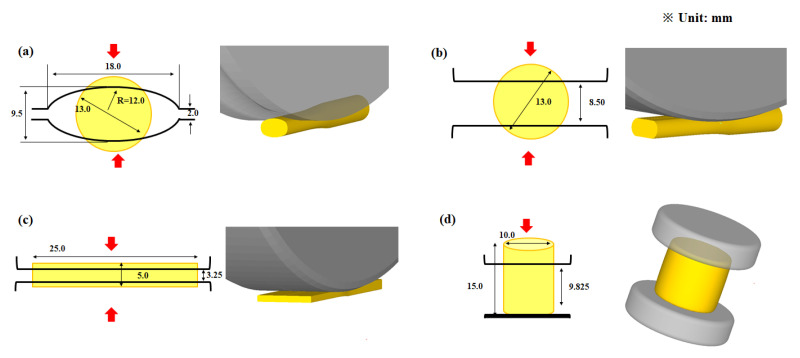
Workpiece and tool geometries for (**a**) rod caliber rolling, (**b**) rod flat rolling, (**c**) plate flat rolling, and (**d**) rod compression.

Several rods with a diameter of 13 mm and length of 400 mm were extracted from the hot-rolled plate using a lathe for conducting the rod caliber rolling and rod flat rolling tests. The cross-section of the workpieces was reduced using grooved and flat rolls with a diameter of 400 mm during rod caliber rolling and rod flat rolling, respectively. The rolling velocity was set to 5 revolutions per minute (RPM) to neglect the influence of temperature increase and strain rate. The initial cross-sectional shape of the workpiece, detailed roll design, and reduction in height (*R_h_*) are presented in Figure 3 and Table 1. The *R_h_* of the workpiece in all processes is calculated as follows:

(1) $$R_{h}=\frac{h_{i}-h_{f}}{h_{i}}\times 100(\%)$$

where *h_i_* and *h_f_* represent the initial and final workpiece heights, respectively. During rod caliber rolling, *h_f_* was obtained by calculating the average roll height due to the oval-shaped roll design. A nominal strain (*ε_n_*) of the deformed workpiece was obtained as follows:

(2) $$\varepsilon_{n}=ln\frac{h_{i}}{h_{f}}$$

For the compression test, the hot-rolled plate was machined into a cylindrical-type compression specimen with a height of 15 mm and diameter of 10 mm. The specimens were compressed using an Instron machine at a rate of 10^−3^ s^−1^ at RT. 

The Vickers hardness (HV) test was carried out with a load of 1.0 kg_f_ and a dwell time of 15 s for the inhomogeneity evaluation of the mechanical properties of the workpiece. 

### 2.2. Deformation Analysis

The plastic deformation of a workpiece is complex during rolling processes, particularly three-dimensional shape rolling such as rod caliber rolling, ring rolling, thread rolling, and rod flat rolling [12,21]. Accordingly, DEFORM 3D software was utilized to analyze the strain, stress, and *P*_c_ distributions in the workpiece during plate flat rolling, rod caliber rolling, rod flat rolling, and rod compression because FEA provides reliable output for 3D complex plastic forming processes. The rolls and dies used in the tests were assumed to be rigid bodies. The workpiece was assumed to be a rigid plastic material because the four workpieces were mainly deformed by plastic deformation. In other words, the average nominal strain of the workpieces was approximately 0.42 during deformation as listed in Table 1; therefore, the elastic deformation can be neglected in this analysis owing to the non-consideration of residual stress. In addition, the workpiece was assumed to be an isotropic material because a hot-rolled specimen was used in this study. The influences of the temperature increase and strain rate during the processes were not considered in this study because of the low forming rate, i.e., the slow strain rate. In such a case, among various constitutive models for material properties, the constitutive behavior of a workpiece can be simply modeled via Hollomon’s law, i.e., *σ* = *Kε^n^* [15,22]. *K* is a constant representing the material strength and *n* is the strain hardening exponent of material. The necessary *K* and *n* values were obtained in the following equation based on the fitting curve in the tensile test, as presented in Figure 2b:

*σ* = 1970.3*ε*^0.535^ (3)

The value of *R*^2^ in this curve fitting was 0.972, which is relatively high and acceptable for modeling the constitutive behavior of a workpiece to simulate plastic deformation of a material. The deviation of the experimental tensile data and fitted curve was relatively high in the elastic region, as shown in Figure 2b, because Hollomon’s law cannot describe the elastic deformation. However, the workpiece was mainly deformed by plastic deformation in these forming processes, as shown in Table 1, indicating that the elastic deformation was not important compared with the plastic deformation. Furthermore, the fitted curve well described the constitutive behavior of a workpiece from a true strain of 0.05 up to a true strain of 0.42, which is the plastic deformation region of this study. 

**Figure 4:**
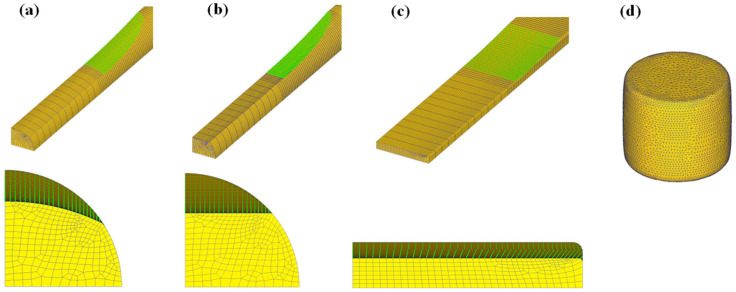
Mesh system for (**a**) rod caliber rolling, (**b**) rod flat rolling, (**c**) plate flat rolling, and (**d**) rod compression.

At the workpiece–tool interface, the shear friction model was applied, and the friction factor was chosen to be 0.3 for all processes [21]. Rods with a diameter of 13 mm were rolled with the 400 mm diameter rolls. The rolling speed was 5 RPM. A quarter of the full geometry was simulated due to the symmetrical process conditions of the four CBFPs used in this study. A hexahedron element was added to all the workpieces, and the number of elements in the workpiece was approximately 16,000. Approximately 250 elements were uniformly put into the cross-section of the workpieces, and 64 elements were inserted along the longitudinal direction of the workpieces, as shown in Figure 4. 

## 3. Validation

**Figure 5:**
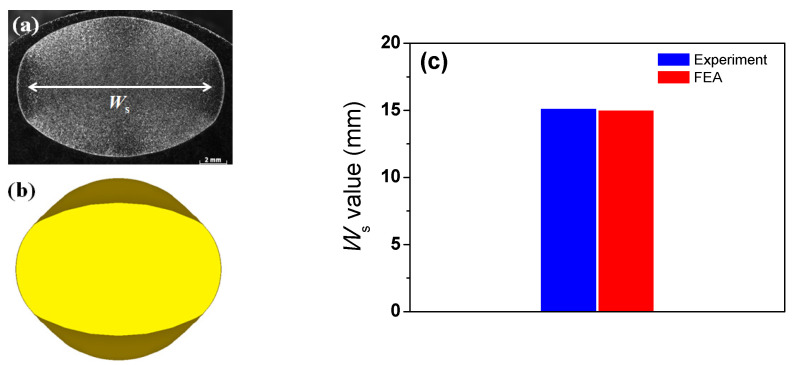
(**a**) Measured and (**b**) simulated cross-section of caliber-rolled rod and (**c**) comparison of lateral spreading of caliber-rolled rod obtained from experiment and FEA.

To validate the present numerical model, the lateral spreading (*W*_s_) results obtained from the experiments and FEA were compared during rod caliber rolling. The *W*_s_ values obtained from the numerical simulation were in good agreement with the measured value, as shown in Figure 5. 

**Figure 6:**
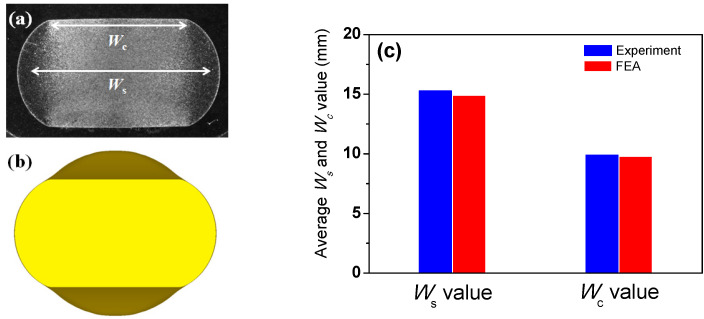
(**a**) Measured and (**b**) simulated cross-section of flat-rolled rod; (**c**) comparison of contact width and lateral spreading of flat-rolled rod obtained from experiment and FEA.

In addition, the contact width (*W*_c_) and *W*_s_ between the experiment and FEA were compared for the rod flat rolling. The *W*_s_ and *W*_c_ values from the FE analysis agreed well with the measured values, as shown in Figure 6, despite a small deviation in *W*_s_ between the two values. Overall, the results of the comparison show that the proposed FEA model is acceptable for further analysis.

## 4. Results and Discussion

### 4.1. Strain Distribution in Workpiece

**Figure 7:**
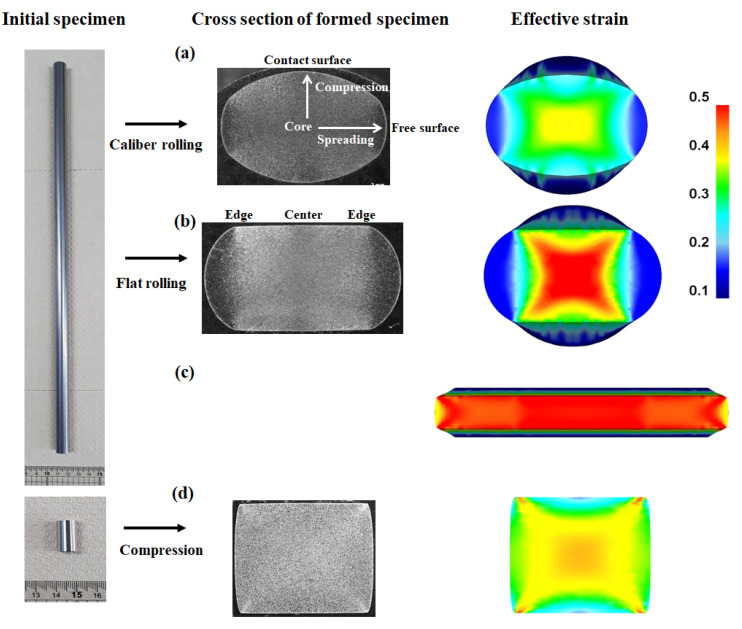
Comparison of shape and effective strain of (**a**) caliber-rolled rod, (**b**) flat-rolled rod, (**c**) flat-rolled plate, and (**d**) compressed rod.

Figure 7 shows a comparison of the cross-section and effective strain (von Mises strain, *ε*_eff_) of the caliber-rolled rod, flat-rolled rod, flat-rolled plate, and compressed rod. The *ε*_eff_ value varied across the workpiece. Except for the flat-rolled plate, the core region of all specimens showed the peak *ε*_eff_, and the surface region exhibited the lowest value. The regions along the diagonal direction showed a relatively high *ε*_eff_, resulting in the occurrence of MSBs. In other words, the regions near the MSBs were harder than the other regions in the workpiece due to the concentration of stress and strain, indicating that the formation of MSBs caused the strain inhomogeneity throughout the region of the workpiece. Eom et al. [9] reported that the peak *ε*_eff_ occurred in the core region of the workpiece during the compression test using steels. In addition, Kazeminezhad et al. [11] showed that the maximum and minimum *ε*_eff_ were developed at the free surface and center regions of the flat-rolled rod based on the copper rod. 

The caliber-rolled rod and flat-rolled rod exhibited the minimum *ε*_eff_ in the free surface, whereas the compressed rod demonstrated the minimum value in the contact surface region. Hwang [16] reported that these results are due to the elongation effect during the rolling process. Meanwhile, the distribution of *ε*_eff_ in the flat-rolled plate significantly differed from that in the caliber-rolled rod, flat-rolled rod, and compressed rod. The contact surface region exhibited the peak ε_eff_, and the core region tended to have low strain in the flat-rolled plate.

**Figure 8:**
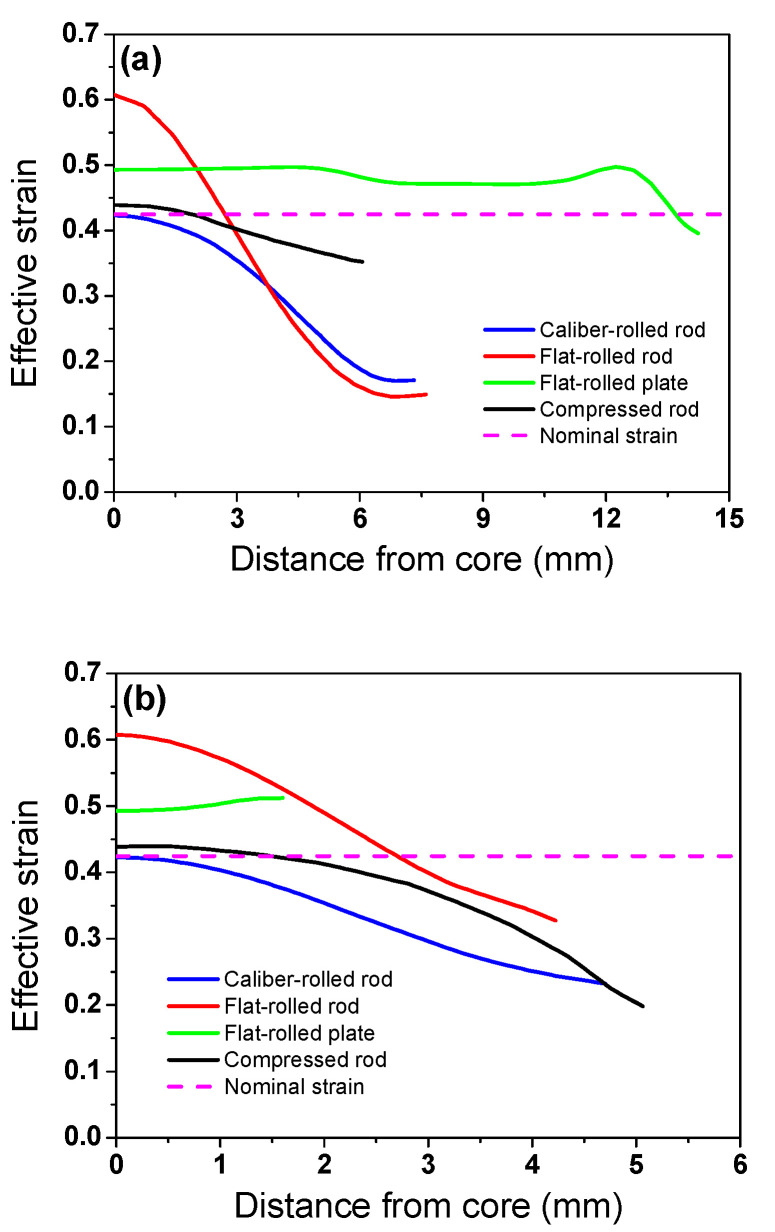
Profiles of effective strain along (**a**) spreading and (**b**) compression directions of four workpieces.

Figure 8 compares the profiles of *ε*_eff_ along the spreading and compression directions of the four workpieces. The highest *ε*_eff_ was observed in the core region of the caliber-rolled rod, flat-rolled rod, and compressed rod, whereas the peak *ε*_eff_ was observed in the contact surface region of the flat-rolled plate. The deviation in *ε*_eff_ was much higher along the spreading than the compression direction in the flat-rolled rod, caliber-rolled rod, and flat-rolled plate. By contrast, in the compressed workpiece, the difference in *ε*_eff_ was higher along the compression than the spreading direction.

**Figure 9:**
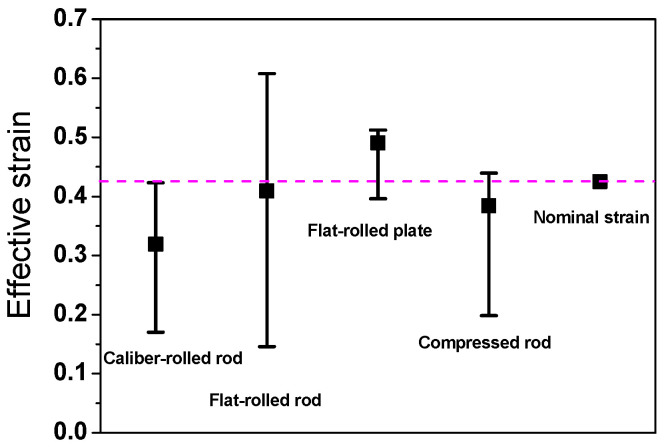
Comparison of average, maximum, and minimum effective strains of four workpieces. Nominal strain was calculated using Equation (2).

Figure 9 shows a comparison of the deviations in *ε*_eff_ of the four workpieces. The flat-rolled plate exhibited a relatively homogeneous strain distribution throughout its region, and the flat-rolled rod demonstrated the maximum inhomogeneity of *ε*_eff_. 

### 4.2. Contact Pressure Distribution on Workpiece

**Figure 10:**
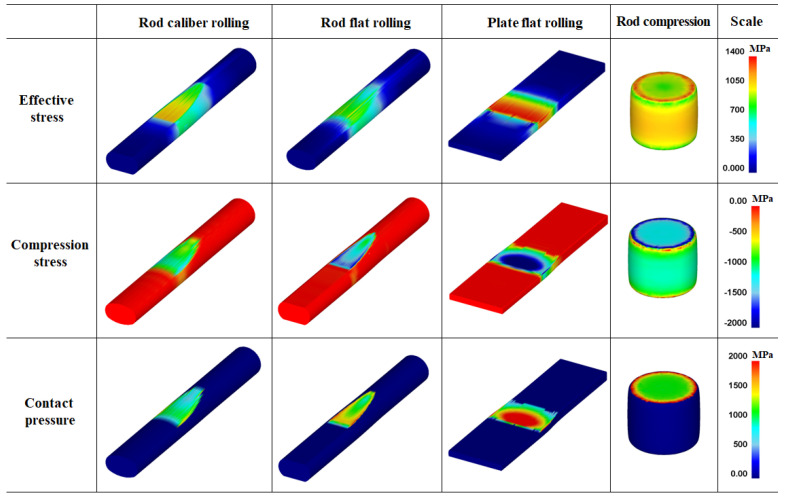
Distributions of effective stress, compression stress, and normal contact pressure on the surface of four workpieces.

**Figure 11:**
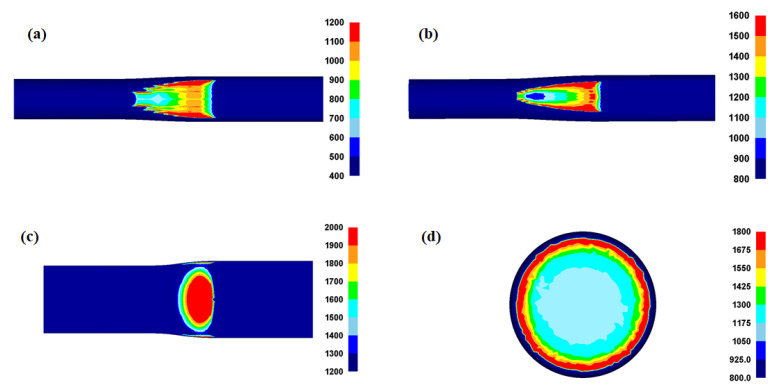
Distribution of normal contact pressure on the surface in a workpiece during (**a**) rod caliber rolling, (**b**) rod flat rolling, (**c**) plate flat rolling, and (**d**) rod compression.

**Figure 12:**
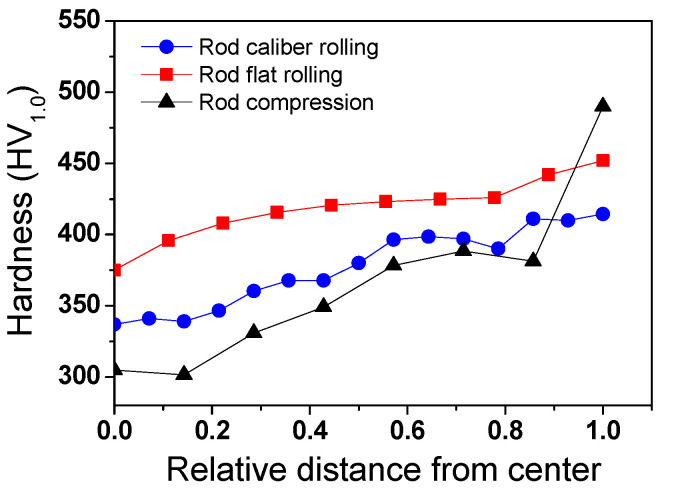
Comparison of the measured average hardness on the contact surface of workpieces along the width direction for different forming processes.

The *P*_c_ and its distribution affect the workpiece shape, forming energy, and temperature distribution of both the workpiece and tool during the forming process [23,24,25]. Figure 10 shows comparisons of the effective stress (von Mises stress), compression stress (downward stress), and *P*_c_ on the surface of the four workpieces. The contours of the effective stress, compression stress, and *P*_c_ differed significantly for the four forming processes. The compression stress was strongly associated with the *P*_c_. Figure 11 compares the *P*_c_ of the four workpieces using different scales for a more detailed analysis. In the case of the flat-rolled rod, the *P*_c_ distribution was high at the entrance zone, edge of the contact region, and exit zone. The *P*_c_ on flat-rolled rods was reported previously [26,27,28]. According to previous investigations, the high value of *P*_c_ in the entrance zone and edge of the contact zone is related to the restrictions caused by the rigid surrounding material, which still undergoes elastic deformation. Additionally, the high value of *P*_c_ in the exit zone is associated with the work hardening rate of the metal during the forming process. The caliber-rolled rod also had *P*_c_ similar to that of the flat-rolled rod. However, the peak strain was not observed at the entrance zone owing to the large contact area between the workpiece and roll at the initial rolling time originating from the grooved oval-shaped roll, as shown in Figure 3. In the compression test, the edge regions of the contact region exhibited higher normal pressure. Interestingly, the distribution of *P*_c_ on the flat-rolled plate was significantly different from that on the other forming processes. The flat-rolled plate exhibited higher normal pressure in the central region. Hartley et al. [29] reported similar results during plate flat rolling. In should be noted that the distribution of *P*_c_ on flat-rolled plates differs with the process conditions, such as the reduction ratio, roll crown, and bending force [30,31]. Figure 12 compares the measured average hardness distribution on the contact surface with the process. The profiles of the measured hardness could reflect the *P*_c_ variations during the process. For instance, the hardness of the center region was lower than that of the edge region in the caliber-rolled rod, flat-rolled rod, and compressed rod. In particular, the deviation in hardness on the contact surface was significantly high in the compressed workpiece because the distribution of *P*_c_ was quite inhomogeneous along the radial direction of the workpiece.

### 4.3. Effect of Contact Pressure on Strain Distribution

**Figure 13:**
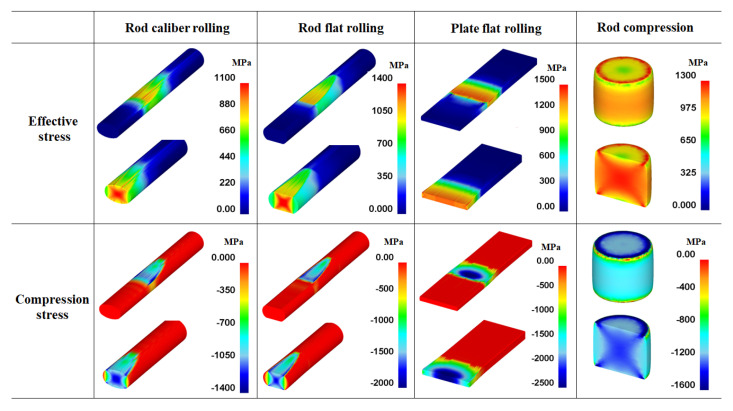
Comparison of effective stress and compression stress of four workpieces.

**Figure 14:**
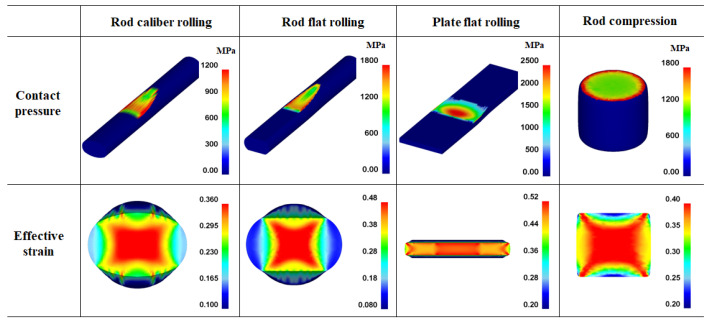
Comparison of contact pressure and effective strain of four workpieces.

The authors believe that the strain inhomogeneity or occurrence of MSBs is influenced by the distribution of *P*_c_. Figure 13 shows a comparison of the effective stress and compression stress on the four workpieces. The pattern of the effective stress and compression stress of the four workpieces was similar. However, the occurrence of MSBs in the cross-sections of the four workpieces was related to the compression stress on the surface of the workpieces rather than the effective stress on the surface. Accordingly, the *P*_c_ and *ε*_eff_ values were compared, as shown in Figure 14, because the distribution of *P*_c_ was strongly related to the compression stress (Figure 10). The distribution of *ε*_eff_ or the strain inhomogeneity depended on the distribution of *P*_c_. The MSBs were stronger as the *P*_c_ was higher in the edge region of the workpiece because the sound metal flow in the width direction of the workpiece was disturbed as the *P*_c_ increased in the edge region. For instance, the flat-rolled rod and compressed rod had strong MSBs due to the higher *P*_c_ value in the edge region. Conversely, the flat-rolled plate exhibited weak MSBs owing to the higher *P*_c_ value in the central region. The MSBs in the flat-rolled rod were stronger than those in the caliber-rolled rod owing to the high *P*_c_ in the edge region of the flat-rolled rod. 

**Figure 15:**
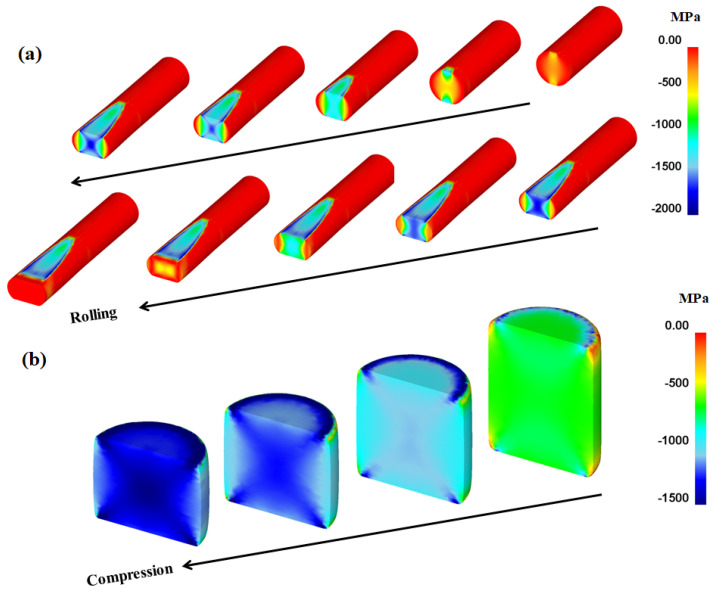
Compression stress variations in workpiece during (**a**) rod flat rolling and (**b**) rod compression with forming steps.

**Figure 16:**
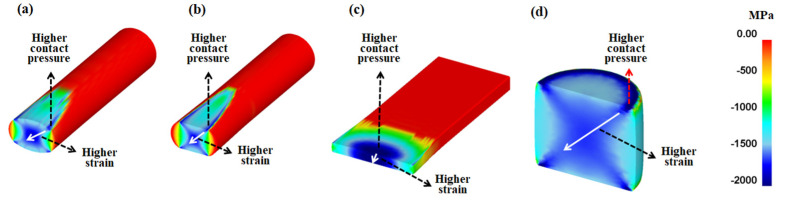
Schematics of effect of contact pressure on the surface on strain inhomogeneity in the workpiece during (**a**) rod caliber rolling, (**b**) rod flat rolling, (**c**) plate flat rolling, and (**d**) rod compression.

In order to confirm the above suggestions, the distribution of the compression stress during the forming steps was examined, as shown in Figure 15. Evidently, the inhomogeneity of the compression stress on the workpiece surface caused product inhomogeneity during the forming steps. The formation of the strain inhomogeneity is schematically shown in Figure 16 from the perspective of the *P*_c_. The results show that stress concentration occurred in the workpiece at the point where the *P*_c_ was relatively high. In order words, the occurrence of MSBs in the workpieces has a strong correlation with the distribution of the compression stress or *P*_c_ on the surface of the workpiece during CBFP.

By understanding the mechanism of MSB generation during CBFP, we can derive conceptual solutions to improve the strain homogeneity of the material properties in the products by controlling the *P*_c_. The *P*_c_ on the workpiece surface should be homogeneous during the forming process by controlling the initial shape of the workpiece, reduction ratio, lubricants, tool design, and temperature of both the workpiece and tool [19,30,31,32]. Cavaliere et al. [30] reported that the distribution of *P*_c_ on the flat-rolled plate varied with the friction coefficient using several numerical simulations. They showed that the homogeneity of *P*_c_ on the flat-rolled plate increased with a decreasing friction coefficient. Their results also indicated that the distribution of *P*_c_ can be controlled using roll design, i.e., roll crown. In this study, the caliber-rolled rod had a smaller peak *P*_c_ compared with the flat-rolled rod, which originated from the large contact area between the workpiece and roll due to the grooved roll shape or caliber roll, indicating that roll design can control the distribution of *P*_c_ on the workpiece during the rolling process. Li et al. [19] revealed that the distribution of *P*_c_ changed from two-peak type to friction hill type with an increasing reduction ratio during the plate flat rolling. And they insisted that the *ε*_eff_ in the specimen with the friction hill type *P*_c_ was more uniform compared with the specimen with two-peak type *P*_c_. This result showed that the distribution of *P*_c_ affected the strain inhomogeneity of material, and it depended on the reduction ratio during the rolling process. Hwang [12] showed that the strain inhomogeneity gradually increased and then decreased with the reduction ratio during rod flat rolling, which means that the maximum strain inhomogeneity occurred at the specific reduction ratio. The author believes that this phenomenon is related to the distribution of *P*_c_ on the flat-rolled rod with the reduction ratio. In addition, Li et al. [19] showed that the distribution of *P*_c_ depended on the strain hardening rate of material based on the comparison simulation of aluminum and copper. The result indicated that the distributions of *P*_c_ and *ε*_eff_ were more uniform with an increasing strain hardening rate of material. It is known that the strain hardening rate of material affects the strain distribution of the workpiece during the metal forming process. Temperature has an indirect effect on the distribution of *P*_c_ during the forming process because the friction coefficient at the material and die interface, and the strain hardening rate and strain rate sensitivity of material, changed with temperature.

Alternatively, the deviation of *P*_c_ on the surface of the workpiece should be reduced by decreasing *P*_c_ in the edge region or increasing *P*_c_ in the central region on the surface of the workpiece. For example, in the case of the flat-rolled plate, the *P*_c_ in the central region of the surface was relatively high, thereby enhancing the homogeneity of the mechanical properties of the workpiece, as shown in Figure 9.

## 5. Conclusions

Based on the comparative study of the distributions of the strain, stress, and *P*_c_ in TWIP steel during rod caliber rolling, rod flat rolling, plate flat rolling, and rod compression, the following conclusions were derived:

1. Strain inhomogeneity or MSBs appeared in the workpiece during the CBFPs. Except for the flat-rolled plate, the core region of all specimens showed peak *ε*_eff_, and the free surface region exhibited the lowest value. The regions along the diagonal direction showed a relatively high *ε*_eff_, resulting in the occurrence of MSBs. However, the level of strain inhomogeneity in the workpiece was different from the forming process, because the formation of MSBs was dependent on the initial workpiece shape and tool design. The flat-rolled rod exhibited the maximum strain inhomogeneity, whereas the flat-rolled plate showed the minimum strain inhomogeneity.
2. The occurrence of MSBs is related to the distribution of *P*_c_ or compression stress. The MSBs were stronger when the *P*_c_ was higher in the edge region of the workpiece because the sound metal flow in the width direction of the workpiece was disturbed as the *P*_c_ increased in the edge region. For example, the flat-rolled plate had weaker MSBs due to the relatively uniform *P*_c_ or higher *P*_c_ value on the central region. By contrast, strong MSBs appeared in the flat-rolled rod and compressed rod because the *P*_c_ value in the edge region of these two processes was high.
3. The strain homogeneity in a workpiece fabricated via CBFP can be improved by tailoring the distribution of *P*_c_ on the surface of the workpiece. To improve the strain homogeneity of the workpiece, the *P*_c_ on the workpiece surface should be homogeneous by controlling the initial shape of the workpiece, reduction ratio, lubricants, tool design, and strain hardening rate of material.

## Figures and Tables

**Table 1:** Process conditions for the CBFPs used in the present research.

CBFP	Height Reduction (*R_h_*, %)	Nominal Strain (*ε_n_*)	Evaluation Method
Rod caliber rolling	35.2	0.43	Experiment and FEA
Rod flat rolling	34.6	0.42	Experiment and FEA
Plate flat rolling	35.0	0.43	FEA
Rod compression	34.5	0.42	Experiment and FEA

## Data Availability

Not applicable.
